# Endometriosis: Epidemiology, Classification, Pathogenesis, Treatment and Genetics (Review of Literature)

**DOI:** 10.3390/ijms221910554

**Published:** 2021-09-29

**Authors:** Beata Smolarz, Krzysztof Szyłło, Hanna Romanowicz

**Affiliations:** 1 Laboratory of Cancer Genetics, Department of Pathology, Polish Mother’s Memorial Hospital Research Institute, Rzgowska 281/289, 93-338 Lodz, Poland; hanna-romanowicz@wp.pl; 2 Department of Operative Gynaecology and Oncological Gynaecology, Polish Mother’s Memorial Hospital Research Institute, Rzgowska 281/289, 93-338 Lodz, Poland; kszyllo@o2.pl

**Keywords:** endometriosis, diagnostic, treatment, biomarkers, genes

## Abstract

Endometriosis is a “mysterious” disease and its exact cause has not yet been established. Among the etiological factors, congenital, environmental, epigenetic, autoimmune and allergic factors are listed. It is believed that the primary mechanism of the formation of endometriosis foci is retrograde menstruation, i.e., the passage of menstrual blood through the fallopian tubes into the peritoneal cavity and implantation of exfoliated endometrial cells. However, since this mechanism is also observed in healthy women, other factors must also be involved in the formation of endometriosis foci. Endometriosis is in many women the cause of infertility, chronic pain and the deterioration of the quality of life. It also represents a significant financial burden on health systems. The article presents a review of the literature on endometriosis—a disease affecting women throughout the world.

## 1. Endometriosis—History

Endometriosis is defined as the presence of the endometrium outside the uterine cavity accompanied by chronic inflammation. This disease was first described by Daniel Shroen in 1690 in the work “*Disputatio Inauguralis Medica de Ulceribus Ulceri*”. The symptoms of this disease were presented by Arthur Duff in 1769 [1].

The first appearances in the literature regarding the pathogenesis of endometriosis appeared in the second half of the nineteenth century. This condition was described by Karl von Rokitansky in 1860, who defined it as the presence of an active endometrium outside the uterine cavity [2].

In 1882, Von Recklinghausen suggested the name *adenomyoma* and by the end of the nineteenth century, several more authors had described this disease.

In 1908, a monograph was published by T.S. Cullen on *adenomyosis* [3].

Cullen was the first to describe the two main symptoms of adenomyosis: prolonged menstrual bleeding and severe pain. He believed that endometrial tissue came from the remains of Müller’s ducts [3,4].

In 1870, the German anatomist, physiologist and pathologist Heinrich Wilhelm Waldeyer was the first to put forward the theory of metaplasia.

One of the outstanding doctors of the nineteenth century, Iwanhofen in 1898 was the author of the thesis that endometrial tissue arises from metaplasia of the peritoneal epithelium.

According to Meyer’s research from 1903, metaplasia would promote the inclusion of the epithelium into the lining, under the influence of hormonal and inflammatory factors.

In Poland, Leonard Lorentowicz was the first to describe endometriosis in Polish Gynecology in 1937 in an article entitled: “On the pathogenesis of intrauterine adenomatosis in the peritoneal cavity (endometriosis peritonealis). Contribution to the etiology of abdominal pregnancy” [4].

Until the 1920s, endometriosis was considered a benefactory hyperplasia disease occurring under various names: cystadenoma, cystic fibrosis, adenomyoma.

In 1927, J.A. Sampson was the first to introduce the term “endometriosis” into medical nomenclature. According to the researcher, the cause of the disease is “retrogradea menstruation” or retrograde transport of menstrual blood with the consequent implantation of exfoliated endometrial mucosa cells within the peritoneal cavity [5,6].

Starting from the first theory of J.A. Sampson, as the years passed and research continued, new theories were formed that attempted to explain the phenomenon of endometriosis. Despite the passage of such a long period of time and much scientific research, Sampson’s theory is still dominant among other hypotheses regarding the etiopathogenesis of endometriosis. It has still not been possible to fully explain why the retrograde transport of menstrual blood, which occurs in nearly 90% of women of childbearing age, only leads to the survival of endometrial tissue outside the uterine cavity in a minority of women [7].

All the theories were sorted out by K. Schweppe in 1984 into three main groups:

- First group—includes theories of endometrial transplantation by retrograde menstrual blood as well as mechanical transplantation (e.g., during surgery), and transport by lymphatic and blood vessels.
- Second group—these are theories of the development of endometriosis in situ; that is, from local tissue from the remains of cells or tissues of the fetal embryo or the genitourinary system. The research of Waldeyer, Russell, Reclinghausen, Gebhard and Meyer and Novak contributed to the above theories [4].
- Third group—includes Levander’s 1941 inductive theory. According to her, exfoliated endometrial cells enter the peritoneal cavity and stimulate its epithelium to transform into endometrial tissue [7,8,9].

Table 1 presents the main theories of endometriosis emerging over years of research on this disease [10,11].

It is worth emphasizing that despite many years of research on endometriosis that tried to explain its etiology and pathogenesis, it still remains an enigmatic disease.

## 2. Endometriosis—Epidemiology 

Endometriosis is characterized by the presence of active foci of the endometrium (glandular cells and stroma) or endometrial tissue (endometrioides; Gr. eides-similar) occurring outside its cavity, that is, in the muscular layer of the uterus, other genitals and their surroundings, and even in places distant from the genital organs of the body [12].

Endometrial foci outside the uterine cavity may appear, for example, in the peritoneal cavity, ovaries, bladder or ureters [13]. The ectopic endometrium is functionally similar to the eutopic endometrium.

Endometriosis is a benign, estrogen-dependent, gynecological disease; however, due to the accompanying ailments and chronic nature, it is a very important medical, social and economic problem.

Infertility is a relatively common symptom in patients with endometriosis. Up to 30 to 50% of women with endometriosis may experience infertility [14]. Endometriosis can influence fertility in several ways: distorted anatomy of the pelvis, adhesions, scarred fallopian tubes, inflammation of the pelvic structures, altered immune system functioning, changes in the hormonal environment of the eggs, impaired implantation of a pregnancy, and altered egg quality. Often, this infertility remains unexplained due to a delay in diagnosis, causing significant levels of stress [15].

Endometriosis is a common gynecological disease in Poland and in the world [16]. This disease affects from 10–15% of women of reproductive age and 35–50% of women with pelvic pain and/or infertility. However, it should be noted that there are also cases of patients with endometriosis after menopause, and it also happens in adolescent women [17].

The vast majority of cases of endometriosis occur in women between menarche and menopause. The peak of the disease falls in the period between 25 and 45 years of age [18].

Literature data indicate that endometriosis is found in 0.1–53% of women operated on laparoscopically or by laparotomy, of which 12–32% are women after diagnostic laparoscopy due to pelvic pain delays and 10–60% of the patient after diagnostic laparoscopy due to disability [19,20,21].

Endometriosis in 7% of women is associated with their genetic predisposition in the family. This disease was found in 2% of women undergoing tubal ligation and 17% of women after surgery to remove the ovaries [22,23].

World literature also reports the occurrence of foci of endometriosis in fetuses [24].

There have been isolated cases of endometriosis in men around the world who have been treated with hormones for prostate cancer [25].

The risk of developing endometriosis is the lowest in black women, the highest in Asian women. Caucasian women have a higher risk of getting sick than black women [26].

Endometriosis is a problem of enormous importance not only from the medical and social angles but also from an economic point of view. The annual costs of endometriosis treatment in Europe range from €0.8 billion to €12.5 billion depending on the country and are comparable to other chronic diseases such as diabetes [27].

Endometriosis has a significant negative impact on aspects of social life, family, and sexual, educational and professional life [27,28,29]. Pain and the associated dysfunction of the body worsen the quality of life and reduce professional productivity. In cases where there is no clear cause or medication, the disease can be chronic and recurrent. Due to its impact on sexuality and fertility, it can have a negative impact on partner relationships.

## 3. Endometriosis—Symptomatology

Endometriosis-related symptoms can affect a woman’s overall health and mental and social well-being. It causes a significant deterioration in the quality of life [30,31]. In 66% of women with endometriosis, the first symptoms of the disease appear before the age of 20 [32]. Symptoms of endometriosis include: gradually increasing acute premenstrual pain, pelvic pain, pain in the sacral region of the spine, dysmenorrhea, painful ovulation, pain during intercourse, pain when defecating, pain when urinating, pain radiating to the back, abundant irregular menstruation, blood in the stool, diarrhea or constipation, infertility and chronic fatigue [18,33].

Patients may also experience uncharacteristic accompanying symptoms such as subfebrile conditions, nausea, dizziness and headaches, symptoms of depression, anxiety, hypoglycemia, rectal bleeding, hematuria during menstruation or susceptibility to infections and allergies.

The pain associated with endometriosis most often takes the form of painful menstruation. It precedes the appearance of bleeding; over time it intensifies and its location concerns the lower abdomen and deeper pelvic areas. Pain can radiate to the sacral region. The pain can extend beyond the bleeding period and also be present throughout the menstrual cycle. There is a hypothesis that the intensification of menstruation soreness is associated with the involvement of the Douglas sinus and the formation of adhesions in it [34].

Sometimes very advanced endometriosis may not cause any symptoms, and, paradoxically, small foci within the peritoneum can cause great pain. Intraperitoneal adhesions or overgrowth of the fallopian gouges are the most common causes of the problem with the treatment of endometriosis. Sometimes foci of endometriosis produce antibodies to the eutopic endometrium, which can induce poor embryo implantation or spontaneous abortions. Increased and profuse menstruation is one of the symptoms of endometriosis, e.g., in adenomyosis (so-called internal endometriosis) [34].

Adenomyosis is defined as the occurrence of ectopic foci of the endometrium outside the uterine cavity. Their effect is lower abdominal pain and abnormal menstrual bleeding. Due to its significant similarity to endometriosis, adenomyosis has so far been classified as endometriosis genitalis interna, in which endometrial foci are located within the muscle membrane of the uterus. In recent years, the distinctiveness of this disease entity has been proven, indicating differences in symptomatology, pathogenesis and treatment [35].

The cause of adenomyosis is still unknown. This disease usually resolves after menopause. The only effective treatment for adenomyosis remains surgery.

The range of diagnosis of adenomyosis varies between 5% and 70% of patients. Adenomyosis is more common in multiparous women than in nulliparous women [36].

The average age of diagnosis of adenomyosis is between 40 and 50 years of age. However, this disease can occur in young women as well as after menopause. The pathogenesis of adenomyosis remains a mystery.

In addition to typical ailments such as menstrual pain, pain during intercourse or pain in the lower abdomen, there are also problems in partner relationships and symptoms of depressed mood [37].

The time from the appearance of the first symptoms of the disease to the diagnosis is up to 8 to 10 years [38].

Despite such frequent occurrence of this disease, the mechanism of its formation remains unexplained, and a good marker of this disease has still not been discovered [39,40].

Deep infiltrating endometriosis (DIE) is defined as the presence of ectopic endometrial tissue infiltration under the peritoneum, pelvic structure, and organ walls, including the uterosacral ligaments, rectosigmoid colon, vagina, rectovaginal septum, bladder, ureter, and lateral parametrium (LP). DIE may cause pelvic pain and thus negatively affect the function of different structures. Studies indicate that women with DIE may have dysfunctions of the pelvic floor muscles (PFMs) and lower limb muscles (LLMs). Pain was associated with PFM hypertonia and difficulty in PFM relaxation [41].

The presence of lateral parametrial endometriosis (LPE) can be considered a reflection of a more severe disease, ureteral stenosis and dilatation, and voiding dysfunctions, mainly because of the involvement of the inferior hypogastric plexus. Patients with LPE reported more frequent constipation and voiding symptoms [42]. Associations exist between LPE and straining to void, the feeling of incomplete emptying, intermittency, and abnormal residual urine and bladder outlet obstruction. LPE might stimulate sympathetic fibers of the pelvic plexus, promoting an increase in urethral sphincter tone and thus leading to different degrees of outlet obstruction. These findings emphasize the importance of obtaining a focused history and objective evaluations of urinary and rectal function in patients presenting with clinical or instrumental findings suggestive of DIE [42].

## 4. Risk Factors for Endometriosis

Risk Factors for Endometriosis Include:

- Early menarche—epidemiological studies analyzing the cycle of women with endometriosis have shown that the early first cycle (before the age of 11) is associated with the risk of endometriosis [43,44,45,46],
- Shorter than 27-day genital cycles, genital defects, including hymen overgrowth or narrowing of the cervical canal [47]. The risk of endometriosis is increased in women with short cycles, i.e., lasting less than 27 days, but is unrelated to the number of bleeding days and the volume of menstruation [48],
- Low BMI,
- Small number of births,
- Caucasian race,
- Age 25–29,
- Daily consumption of alcohol in the amount of at least 10 g per day,
- Endometriosis is more often diagnosed in infertile women who are active smokers and whose body mass index (BMI) is normal or low [49].

Interestingly, the latest data indicate that there is generally no association between BMI and the incidence of endometriosis, but there has been a significant increase in the incidence of endometriosis in obese women compared to women with normal body weight. Obesity is also a risk factor for severe dysmenorrhea [50].

Diet plays a very important role in preventing the development of endometriosis. The consumption of green vegetables and fresh fruits is considered to be the most beneficial [51]. They contain antioxidants, which play an important role in the proper functioning of the immune system and the removal of free radicals. It is worth noting that the fiber contained in vegetables interacts in the control of the intestinal bacterial flora and affects hormonal balance.

Red meat exerts an antagonistic effect on the development of endometriosis compared to vegetables and fruits. It is characterized by a high content of dioxins, hormones and fat, increasing the concentration of estrogens [51].

In the latest research by the team of Yamamoto et al., an attempt was made to determine whether higher consumption of red meat, poultry, fish and seafood is associated with the risk of laparoscopically confirmed endometriosis [52]. The study group consisted of 81,908 women, and the observations covered the years from 1991 to 2013. Diet was assessed using a properly prepared nutrition questionnaire administered every 4 years. It was shown that respondents who reported eating > 2 servings/day of red meat had a 56% higher risk of endometriosis compared to those consuming ≤ 1 serving/week. Women who were classified in the category that consumed the most red meat were more likely to have endometriosis. No association with poultry, fish, shellfish and egg consumption and the risk of endometriosis was demonstrated [52].

A very important factor in the prevention of primary endometriosis is the maintenance of an appropriate lifestyle, in which a significant part should be occupied by rest, movement and physical activity [51].

## 5. Theories of the Formation of Endometriosis

So far, the pathomechanisms of the formation of endometriosis have not been definitively explained. The theory of Samson (“retrograde menstruation”) is more widespread. It says that the foci of endometriosis arise as a result of the displacement of menstrual blood into the peritoneal cavity through the fallopian tubes [5,6,53].

Literature data indicate that in 80% of women with open fallopian tubes there is a retrograde outflow of menstrual blood, while endometriosis occurs only in some. This suggests the presence of other factors determining the survival of endometrial cells in the peritoneal cavity and their implantation [54].

Implantation in the peritoneum is explained by a local disorder of the mechanisms that prevent adhesion. The consequence is an increased production of cytokine macrophages, including tumor necrosis factor α (TNF-α) and interleukin [55].

Immune phenomena are important for explaining the possible pathomechanisms of endometriosis foci, as studies indicate altered humoral and cellular immunity [56].

There are literature data on the statistically higher occurrence of certain immune diseases together with endometriosis, for example, rheumatoid arthritis or hypothyroidism [57]. Another way of creating endometriosis foci was presented by Mayer’s theory, which says that peritoneal cells are transformed into Muller-type cells under the influence of hormones [58]. This theory is based on the assumption of the existence of cells capable of differentiating in the endometrium and those cells being precursors of the mesodermal epithelium of the ovary and the pelvic peritoneum. This theory is particularly useful in explaining the existence of endometriosis in different regions of the body where there is a mesothelium, e.g., pleural cavity [59,60].

The last theory is, in the light of two previous theories, that endogenous biochemical and immunological factors responsible for the non-functioning of endometrial factors are not being used for endometrial systems [61].

Endometriosis is also considered a chronic inflammatory process associated with immune processes [62]. Disorders of the immune system accompany virtually every stage of endometriosis development [63]. Macrophages are known to participate in recognizing foreign cells and presenting them to T cells. An increased number of activated macrophages with a reduced ability to phagocytose in the peritoneal cavity is characteristic of women with endometriosis.

They secrete pro-inflammatory cytokines, such as IL-6, TNF-α, IL-1β and IL-8, in increased amounts. Peritoneal macrophages in women with endometriosis have increased mRNA expression of the cyclooxygenase-2 (COX-2), which resulted in increased prostaglandin secretion [64]. Increased release of pro-inflammatory cytokines and reduced production of anti-inflammatory factors from stromal, epithelial, smooth or immune cells contribute to the initiation, development and progression of endometriosis [65] (Table 2).

Another important factor in the pathogenesis of endometriosis is the disturbed balance between type 1 (Th1) and type 2 (Th2) helper lymphocytes. Activated T cells differentiate into Th1 lymphocytes and Th2 lymphocytes. The main function of Th1 is the production of cytokines and the promotion of cell-type responses, whereas Th2 secretes cytokines involved in the differentiation of B lymphocytes, suppression of cell-type responses, as well as the severity of humoral type responses. According to literature data, Th2 lymphocytes gain an advantage in women with endometriosis [79].

In women with endometriosis, reduced Natural Killer (NK) cell activity is found. This is the main group of cells of the immune system responsible for the phenomenon of natural cytotoxicity. Impaired function of NK cells reduces their ability to cleanse the peritoneal cavity of endometrial elements after retrograde outflow of menstrual blood [80].

The formation of new vessels is a necessary condition for the development of the ectopic endometrium, especially in the peritoneal microenvironment. Neo-angiogenesis is accompanied by the formation of nerves, which may explain the pain in patients [81].

Vascular endothelial growth factor (VEGF) is responsible for the formation and growth of new vessels. In women with endometriosis, elevated concentrations of VEGF in peritoneal fluid and its correlation with the stages of the disease were found [82].

At the moment, scientists agree that many factors are responsible for the formation of endometriosis, and in particular genetic, immunological and environmental factors [83].

It has been confirmed that genetic conditions may be factors influencing the development of this disease. Currently, a group of genes that can enable the formation of endometriosis is listed. These include the cytochrome P450 gene, estrogen, progesterone and androgen receptor, as well as the p53 gene [83].

## 6. Types of Endometriosis

There are several types of endometriosis:

- Ovarian endometriosis—occurs in the form of superficial lesions and as endometrial cysts,
- Peritoneal—can occur in various forms: white raids on the peritoneum, peritoneal defects, red, brown, black-blue and black foci, colorless bright vesicles and focal dilated blood vessels and petechiae,
- Deep infiltrating endometriosis—DIE,
- Endometriosis of other locations.

The three most typical types of endometriosis are peritoneal endometriosis, ovarian cysts (chocolate cysts) and nodules of deeply infiltrating endometriosis in the gut or vaginal–rectal septum [84].

Peritoneal endometriosis can occur in the form of intraperitoneal and sub-peritoneal. Foci of endometriosis within the peritoneum are found in 15–50% of all women diagnosed with endometriosis [85]. The best diagnostic method for detecting these changes is to recognize them during laparoscopic surgery [86].

Ovarian endometriosis occurs in 2–10% of women of reproductive age and 50% of patients treated for infertility [87]. Ovarian endometriosis is one of the most common localizations of this condition.

Deep infiltrating endometriosis is characterized by the fact that endometrioid changes can reach deep into the extraperitoneal space and occupy various pelvic organs such as the bladder, ureters, large intestine, sacro-uterine ligaments or the vagina. The pathomechanism of DIE is not clearly defined.

## 7. Classifications of Endometriosis

In order to assess the location and severity of the disease, many divisions and classifications have been proposed.

According to Sampson [88], we will divide it into:

- Internal endometriosis affecting the uterine muscle
- External endometriosis occurring outside the uterine muscle

Divisions of endometriosis by location were developed according to the classification of Martius and Kistner [89,90] (Table 3).

Based on the histological classification according to Brosens (1993), we can distinguish types of endometriosis [91]:

1. Mucosal type (occurs in endometrial cysts of the ovary),
2. Operitoneal type (exhibits multi-focus and morphological diversity)
  - early, active, glandular or follicular lesions,
  - advanced, black, wrinkled changes,
  - white fibrotic lesions,
3. Glandular type (the main element is fibrous-muscular tissue and concerns deeply infiltrating endometriosis).

Among the many divisions of endometriosis according to the extent and severity of lesions, the division developed by the American Fertility Association (AFS) is the most commonly used. The American Society of Reproductive Medicine distinguishes four stages of endometriosis, where stage I and II are fairly mild types, and stages III and IV are advanced disease [92,93]. Currently, there is a division corrected by AFS in 1985.

The classification developed by the American Society of Reproductive Medicine (ASRM), based on the results of laparoscopy or laparotomy, it is the most common system used in clinical practice. The most important goal of classifying and determining the severity of endometriosis is to propose an effective treatment plan for it [94].

The ENZIAN scale in deeply infiltrating endometriosis is a descriptive scale, considering both the existence of the lesion and the depth of the invasion. In the ENZIAN classification, the location of foci was assigned to separate anatomical compartments [95].

- Compartment A—foci located in the vagina and the rectovaginal septum,
- Compartment B—foci located in the sacro-uterine ligaments up to the pelvic walls,
- Compartment C—foci located in the sigmoid colon and rectum.

This classification also describes the foci of the ectopic endometrium depending on the place of their occurrence as:

FA—adenomyosis,
FB—urinary bladder endometriosis,
FU—ureter endometriosis,
FI—endometriosis of the bowel wall above the sigmoid colon,
FO—infiltration of other anatomical structures, e.g., abdominal integuments.

Endometriosis occurring outside the pelvis is a rare phenomenon. However, literature data provide information on respiratory endometriosis or pericardial endometriosis or endometriosis in a scar after surgery with laparotomy access [96,97].

## 8. Diagnostics

Histopathological examination clearly allows for the diagnosis of endometriosis. However, a good medical history, gynaecological examination with specula, two-handed examination, additional diagnostic tests using imaging techniques, laparoscopy and biochemical tests are helpful in the initial diagnosis of the disease.

The basic examination in the diagnosis of endometriosis is an ultrasound examination [98]. Ultrasound examination (ultrasonography, USG) is helpful in the diagnosis of endometrial cysts of the ovary and of congenital defects of the reproductive organs favoring the retrograde outflow of menstrual blood into the peritoneal cavity [99]. In the case of endometriosis infiltrating the urinary bladder or the large intestine, it is justified to perform cystoscopy, colonorectoscopy and transrectal ultrasound examination [99].

**Figure 1:**
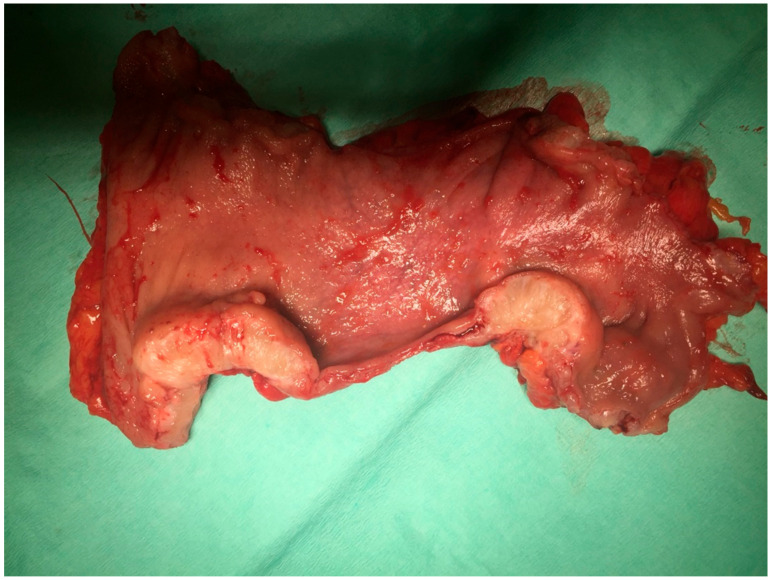
Intestinal endometriosis (from Department of Operative Gynaecology and Oncological Gynaecology, Polish Mother’s Memorial Hospital Research Institute, Lodz, Poland).

In the case of deeply infiltrating endometriosis, the Rectal Water Contrast Transvaginal Sonography (RWC TVS) is also appropriate. The water contrast allows us to detect foci in the intestinal area and assess their progression (Figure 1).

Deeply infiltrating endometriosis is characterized by the infiltration of endometrial cells > 5 mm below the surface of the peritoneum [100]. Profound lesions mainly affect the posterior pelvic compartment, including the rectovaginal septum, posterior vaginal vault, utero sacral ligaments and anterior rectal wall, causing adhesions and distortions of pelvic anatomy [101,102]. In addition to the classic DIE pain syndrome (characterized by dysmenorrhea, dyspareunia, chronic pelvic pain, dystrophy and dyschesia), profound changes are associated with dysfunction of the pelvic organs and pelvic floor muscles (PFM) [103]. A series of events or a combination of factors may contribute to the development of non-relaxing PFM in women with chronic pelvic pain, including direct or indirect (neuropathic) pelvic floor muscle injury, pelvic pain symptoms and inflammation. Evaluation of PFM by palpation or electromyography can cause pain, causing pelvic muscle spasm, which can be a confounding factor. Transperineal ultrasound has been shown to be an important, reliable and non-invasive tool for assessing pelvic floor morphometry [104,105]. Women with DIE have a smaller area of the levator hiatal area (LHA), and dynamic maneuvers with three-dimensional (3D) and four-dimensional (4D) transperineal ultrasound suggest they have higher muscle tone and lower strength. [106] than those without DIE. In a 3D/4D transperineal ultrasound examination, women with ovarian endometriosis and concomitant profound lesions appear to have increased PFM tone and decreased PFM strength compared to women with isolated ovarian endometriosis [107]. Transperineal ultrasound is a reliable and non-invasive tool for assessing pelvic floor morphometry [108]. Pelvic floor hypertonic dysfunction is a complex condition, and the specific cause or event that triggers this disorder is often not clearly identified [109]. A higher tone of PFM is associated with its damage and innervation (i.e., surgery, trauma or endometriosis), musculoskeletal changes (i.e., skeletal asymmetry), chronic pelvic pain syndromes and/or dyspareunia [110,111].

Moreover, this condition can also be attributed to abnormal learned pelvic floor activity or poor activity acquired in adulthood, through constant voluntary urination or defecation, or through sexual abuse [112]. The relationship between hypertonic disorder PFM and DIE can be explained by two main mechanisms. First, women with ovarian endometriosis alone tend to have less severity of pain symptoms than women with peritoneal endometriosis. In addition, in DIE lesions, increased density of nerve fibers and the phenomenon of perineural and intraneural invasion were clearly demonstrated [113]. Women affected by DIE showed smaller LHA than women with isolated ovarian endometriosis. Hypertonic dysfunction of PFM may be associated with pain symptoms (myofascial pain) and changes in pelvic organ function in women with DIE. Indeed, the role of PFM is not only to support the pelvic organs, but its contraction and relaxation are necessary for the proper functioning of the intestines and bladder and to enable painless sexual intercourse. While hypotonic pelvic floor disorders, such as pelvic organ prolapse, are often easily identified, symptoms of hypertonic PFM disorders are often not well studied, making these disorders unreported or misdiagnosed as other, better known concomitant conditions [109]. Because PFM dysfunction can cause pain symptoms and pelvic organ dysfunction potentially resistant to hormonal or surgical endometriosis therapy, transperineal ultrasound may enable a more complete functional assessment in women with DIE, with the goal of achieving tailored therapy, including pelvic floor rehabilitation.

It is also helpful to have a magnetic resonance imaging (MRI) examination, but the ultrasound examination is the basic tool in the diagnosis of this disease.

However, the gold standard in the diagnosis of endometriosis is laparoscopic surgery, with simultaneous confirmation in histopathological examination [114].

The less frequent areas of endometriosis include the vaginal vault and cervix, which is why examination with a speculum is an obligatory element of the gynaecological examination. Palpation can detect endometriosis in the area of the vaginal-rectal septum and the sacro-uterine ligaments. A characteristic and frequently encountered feature in patients with endometriosis is also uterine retroflexion, often forced by intraperitoneal adhesions.

## 9. Treatment

Treatment of endometriosis is a big medical problem due to the fact that this disease is difficult to treat and is chronic in nature. Pharmacological, surgical or combination treatment is possible.

Symptoms associated with the presence of foci of endometriosis are the main factor determining the need to start treatment. These symptoms are pain complaints of varying severity and location, and problems with getting pregnant. Women who notice abnormalities in their body report to the doctor, which allows them to apply a selected treatment in advance. A big problem remains patients who, despite the occurrence of pain, report late and therefore the disease, especially DIE, causes the involvement of other organs and the need to perform extensive complicated surgical procedures. Pharmacological, surgical and combination treatments are used to treat endometriosis [115]. In pharmacological treatment, we distinguish between hormonal and symptomatic treatment. Currently, hormonal treatment is allowed, for three months without histopathological confirmation of the disease. The choice of treatment is made depending on the age of the patient and her procreative desires, the ailments present and the form of endometriosis. Proper diet and lifestyle also play an important role.

### 9.1. Pharmacological Treatment

Drug treatment is used in women in their reproductive years. The goal of pharmacological treatment is to reduce or eliminate pain, inhibit further development and regression of endometrial foci and restore fertility. Initiation of pharmacological treatment of endometriosis is possible only on the basis of a clinical exam without the need to confirm the existence of the disease in laparoscopic examination (empirical therapy).

Pharmacotherapy may be part of the preparation for surgery, as well as a complementary procedure in the postoperative period. There are certain types of drugs that are used in the treatment of endometriosis (Table 4).

Non-steroidal anti-inflammatory drugs inhibit the synthesis of prostaglandins, contribute to reducing the inflammatory process and resolve pain [114]. Complex estrogen-progestogen therapy can be used cyclically or continuously.

In hormone therapy of endometriosis, the following groups of drugs are used [116]:

Danazol—a derivative of the male sex hormone testosterone. It inhibits the secretion of GnRH, causing a decrease in the secretion of LH and FSH by the pituitary gland. Therapy is long-term (6–9 months) and is started on the scond day of the cycle. The drug has many side effects. These include weight gain, fluid retention, breast reduction, oily skin and acne, hot flashes, decreased voice timbre.

Gonadoliberin analogues are substances with a structure similar to GnRH, which “deceive” the hypothalamus, causing a decrease in the production of real GnRH. This results in a decrease in LH and FSH levels and a significant reduction in estrogen levels. Therapy lasts at least 3–6 months. Side effects are caused by low estrogen levels. These include loss of calcium from the bones leading to osteoporosis, vaginal dryness, decreased libido, headaches, insomnia.

Progestogen preparations—contain progesterone derivatives, whose function is to reduce the level of GnRH, and thus the levels of FSH, LH and estrogen. Therapy should be used for at least 6 months. The most common side effects include fluid retention, weight gain, breast tenderness, spotting and irregular bleeding from the genital tract.

Oral contraceptives (estrogen-progestogen preparations)—their action consists in lowering the level of FSH and stabilizing the endometrium, which leads to a reduction in pain.

An intrauterine device releasing levonorgestrel is a progesterone derivative. The effect is the same as progestogen preparations. Recommended especially for women with severe pain.

Aromatase inhibitors—the latest group of drugs used in the treatment of endometriosis. Their action takes advantage of the fact of specific behavior of endometriosis cells. Foci of endometriosis, regardless of the ovaries, produce estrogens that cause an increase in endometrial lesions. Aromatase inhibitors interrupt estrogen production in both endometriosis foci and ovaries, causing a significant reduction in estrogen levels. Side effects are due to low estrogen levels. The most important of these is the significant loss of calcium from the bone causing osteoporosis. Others include loss of libido, vaginal dryness, insomnia [116].

All recognized methods of pharmacological treatment have similar therapeutic effectiveness. They differ in the scope and speed of the appearance of side effects. There is still an urgent need to develop new types of pharmacological therapies that will guarantee a complete cure of patients without the occurrence of side effects.

The latest drug to be approved by the Food and Drug Administration (FDA) is elagolix, a drug for the treatment of moderate to severe pain associated with endometriosis [117]. The results of a study in which about 1700 women with moderate or severe pain in endometriosis participated played a part in the above decision. Doses of this drug—150 mg once a day or 200 mg twice a day—significantly reduced the most common types of endometrial pain: pelvic pain and sex-related pain. For a dose of 150 mg, the duration of use of the drug is 24 months; for a dose of 200 mg duration is limited to 6 months, as the drug causes a decrease in bone mineral density. The drug is taken orally [117].

Elagolix is a non-peptide GnRH (gonadotropin-releasing hormone) antagonist. Elagolix enables dose-dependent reductions in estrogen levels and is effective in relieving moderate to severe endometriosis pain with long-lasting, sustained effects. As a GnRH antagonist, elagolix may also reduce bone mineral density through its estrogen reduction mechanism [118].

As evidenced by literature data, although there is a risk of bone loss when elagolix is used, the effect of elagolix on the long-term risk of fracture is minimal [119,120].

Results from the final model simulations indicate support for recommendations for the use of elagolix at a dose of 150 mg q.d. for 24 months [121].

### 9.2. Surgical Treatment

In the case of surgical treatment, it can be sparing or radical. Sparing treatment applies to adolescent patients and women of childbearing age planning to become pregnant. Radical surgical treatment is carried out in patients who do not plan pregnancy or those who continue to have pain despite the pharmacotherapy used.

Indications for surgical treatment of endometriosis are as follows:

- pelvic pain,
- infertility in endometriosis,
- endometrial ovarian cysts.

Laparoscopy is the recommended surgical technique for the treatment of endometriosis, regardless of its stage [122]. The best therapeutic effects are achieved as a result of the combination of surgical treatment with pharmacological treatment. Medications are applied both before and after surgery.

Laparoscopy with the assistance of a robot is a viable method of resection of deeply infiltrating endometriosis, especially in the rectal-sigmoid region. In the case of endometriosis, a mini-invasive laparoscopic or laparoscopic approach using a robot is strongly recommended [108]. The disadvantage of surgery, however, is that when removing DIE lesions, complications that affect the functions of the gastrointestinal, urinary or sexual tract often arise. Complications after DIE surgery include rectal fistula (0.3–2%), intestinal stenosis (2%), and bladder atony (4–6%) [123,124,125].

Several studies have compared laparoscopic or robotically assisted methods in the surgical treatment of endometriosis [126,127,128].

Laparoscopically assisted robotics are associated with longer duration of relief than laparoscopic surgery, but the results are controversial [129,130].

The results of previous studies on the benefits of robot-assisted laparoscopy compared to conventional laparoscopy are somewhat heterogeneous. However, patients with features of a complex pelvic situation, such as severe endometriosis, increased body mass index, or previous surgeries, may benefit from robotic surgery [131].

Endometriosis treatment between robot-assisted laparoscopy and conventional laparoscopy was compared in [130].

This study did not detect any differences in perioperative outcomes of surgery between robotic surgery and laparoscopy.

The results of other smaller studies, mainly retrospective, were heterogeneous. Some of these studies have shown longer surgery times for robotic assisted procedures than for laparoscopy, whereas other studies have shown benefits of robotic surgery [132].

Laparoscopic rectal nodulectomy with nerve-sparing robots has been shown to be a feasible and safe treatment for isolated sting-rectal DIE [133].

The latest study suggests that robot-assisted laparoscopy is a possible method of DIE resection [134].

### 9.3. Physiotherapy in Endometriosis

Physiotherapy in endometriosis focuses on non-invasive and conservative treatment of pelvic floor disorders in women. It deals with the restoration of the efficiency and function of tissues and organs in the pelvic area, supports the process of surgical treatment, relieves pain, thus improving the quality of life [135].

In women suffering from endometriosis, pelvic floor structures are very often dysfunctional. Therefore, it is an indication for urogynecological physiotherapy. Inflammation accompanying recurrent endometriosis can cause tissue damage, and thus the formation of scar tissue to heal the affected areas. Unfortunately, a side effect of this process is the formation of adhesions, which, depending on the location, can cause different symptoms. Occurring in the abdominal cavity, they can limit the mobility of internal organs and contribute to the formation of symptoms, i.e., flatulence, constipation, digestive problems, problems with maintaining proper body posture and pain. For such ailments, it is a good idea to use manual therapy and osteopathic techniques to improve the mobility of internal organs and structures of the pelvis and spine. The following types of techniques can be successfully used: mobilization of the lumbosacral spine, needle therapy, pinopressure, visceral therapy (general abdominal maneuvers, therapy of female organs, intestines, liver), if necessary and with the consent of the patient of the technique per vaginum. As a consequence, in combination with the classic treatment of endometriosis and urogynecological physiotherapy, the course of the disease may become much milder and will allow the patient to return to everyday life [135].

Physical therapy techniques are known to reduce pain and improve quality of life in endometriosis. The overall goal of treatment is to teach the patient to relax the muscles, which in turn helps to break the pain cycle [136].

Evidence suggests that the symptoms of endometriosis are the result of a local peritoneal inflammatory response triggered by an ectopic endometrial implant that undergoes cyclic bleeding [137].

Regular exercise, on the other hand, has a protective effect against inflammatory diseases because it induces an increase in systemic levels of cytokines with anti-inflammatory properties [138].

Regular exercise is thought to promote reduced menstrual flow, ovarian stimulation, and estrogen effects [139].

Analysis of the available literature data shows that there are no controlled and randomized trials determining whether and to what extent exercise can be beneficial for women with endometriosis. So far, researchers have only speculated on this subject [140].

Physical exercise has been shown to have a beneficial effect on muscle relaxation in patients suffering from endometriosis, which in turn helps to break their pain cycle [141].

Progressive muscle relaxation training has been shown to be more effective in reducing pain, anxiety, and depression in women with endometriosis undergoing hormone therapy [142].

Cardiovascular activity helps endometriosis patients maintain good energy levels. Exercise is one of the most effective strategies for increasing serotonin levels; physical activity and deep breathing exercises can increase the rate of burning serotonin neurons in the brain, which can stimulate the production of mood-enhancing substances. Aerobic exercise, such as walking and swimming, can have a more significant effect on serotonin levels, strengthening the muscles of the whole body and improving overall circulation [143].

However, there are literature data that draw attention to the possibility of inferring the non-protective effects of exercise in women with endometriosis, which may result from the discomfort felt that prevents physical exercise [141,144].

## 10. Biomarkers

**Figure 2:**
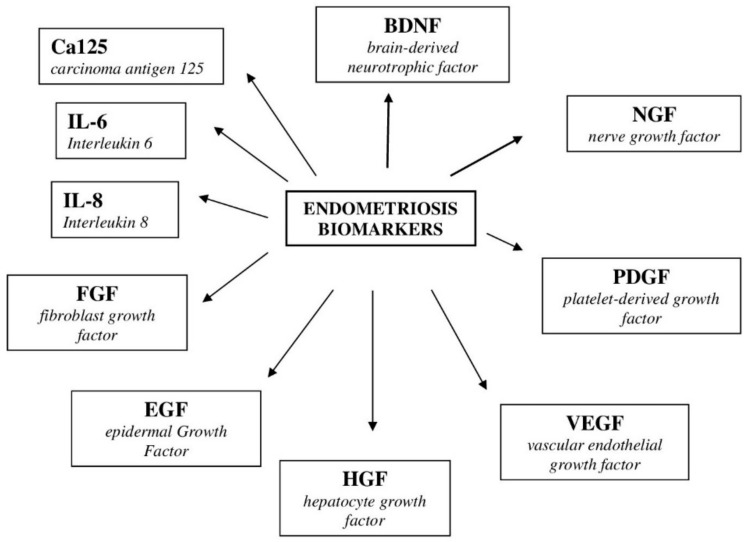
The potential endometriosis biomarkers.

So far, no specific marker for endometriosis has been identified. The search is underway for a biomarker obtained from serum, plasma or urine, which would allow for a quick and simple diagnosis. In recent years, many substances have been tested that could be potential markers for endometriosis (Figure 2). With peritoneal endometriosis, serum and plasma are not good diagnostic material; attention is paid to the eutopic endometrium. Eutopic endometrial tissue is available by taking the endometrium through a biopsy.

The endometrium comes from the intermediate mesoderm during mesenchymal-epithelial transition (MET) during the formation of the genitourinary system. It is a reversible process, therefore, during the epithelial-mesenchymal transition (EMT, reverse process MET), it is possible to reach the formation of foci of endometriosis [145]. Foci of endometriosis behave in the same way as eutopic endometrium. There is monthly bleeding within them, which trigger an immune response. This causes the foci of endometriosis to increase [146].

Pain is a typical symptom of endometriosis, so the conduction pathways were analyzed and the brain-derived neurotrophic factor (BDNF) was found to be elevated in women with endometriosis compared to the control group [147]. BDNF is a protein that affects sensory neurons. As research shows, BDNF is not useful in detecting peritoneal endometriosis and deeply infiltrating endometriosis [148].

The marker of endometriosis was sought among growth factors and cytokines, because the development of ectopic implants is accompanied by the processes of invasion, proliferation and angiogenesis. The most promising results were obtained for hepatocyte growth factor (HGF), fibroblasts growth factor (FGF), epithelial growth factor (EGF), vascular endothelial growth factor (VEGF) and interleukin IL-1, IL-6 and IL-8. The most reproducible results were obtained for IL-6. IL-6 is the most characteristic of endometriosis. Its sensitivity is 63% and its specificity is 69% [149].

YKL-40, a new biomarker of inflammation, is secreted by activated macrophages and neutrophils in various inflamed tissues. Serum concentrations of YKL-40 are elevated in patients with diseases characterized by inflammation. Protein YKL-40 (CHI3L1–chitinase-3-like protein 1, HC-gp-39–human cartilage glycoprotein-39, gp-38k, chondrex) is the subject of scientific research looking for new biomarkers of endometriosis. Literature data suggest that circulating YKL-40 levels may be a new biomarker for diagnosing and monitoring endometriosis. The studies established serum concentrations of YKL-40 in patients with endometriosis compared with healthy people of the same age. It was shown that the level of YKL-40 was significantly higher in patients with endometriosis compared with healthy women [150].

It is known that in places affected by endometriosis, oxidative stress further intensifies the immune response, as a result of which there may be an exacerbation of the development of endometriosis [151,152,153]. Women with endometriosis have increased oxidative stress in the peritoneal cavity. Data indicate that oxidative stress occurs mainly in the peritoneal cavity in women with advanced stage of the disease [154].

Polak et al. showed that the peritoneal fluid of women with endometriosis is characterized by impaired iron metabolism [154]. This is most likely related to the increased number of erythrocytes in the peritoneal cavity of women with endometriosis, which leads to a higher haemoglobin concentration in this environment. Impaired iron homeostasis can have a significant effect on the pathophysiology of peritoneal endometriosis through the direct influence of haemoglobin derivatives and/or the creation of a pro-inflammatory and pro-oxidative environment.

Oxidative stress has been shown to activate the transcription factor NF-κB. The NF-kB protein family consists of the proteins RelA (p65), RelB, c-Rel, p50 and p52. The active transcription factor always consists of two subunits and the most common variant of NF-kB is the RelA (p65) and p50 complexes. NF-kB contains a signalling sequence that allows the transport of the active complex to the cell nucleus and binding to a specific DNA consensus. In an inactive form, the NF-kB factor is localized in the cytoplasm of the cell in association with the inhibitor protein IkB. Activation of the transcription factor NF-kB occurs through the interaction with the receptors of the cells of pro-inflammatory cytokines (e.g., TNF-alpha, LPS or IL-1beta), but also such factors as UV rays, antigens or free radicals. Activation of the specific receptor subsequently leads to phosphorylation of the inhibitory IkB protein by IKK kinases followed by proteosomal degradation of IkB. The released complex can be translocated to the cell nucleus [142]. This pathway of activation of the NF-kB factor is referred to as the canonical pathway. In addition to the canonical NF-kB activation pathway, the action of LPS, CD40 or lymphotoxin (TNFbeta) may lead to the so-called non-canonical NF-kB activation pathway. The transcriptional activity of NF-kB is crucial for cell function and proliferation. The elevated concentrations of TNF-alpha, TNF-beta, LPS, IL-1beta and CD40 in peritoneal fluid in women with endometriosis promote processes such as adhesion, invasion, proliferation and angiogenesis that stimulate the development of the disease [155].

Unfortunately, despite the passage of years and many studies in which an endometriosis marker was sought, none of the tested substances can be recommended for the diagnosis of endometriosis.

The only markers that have found partial clinical use in the diagnosis of endometriosis are the glycoproteins Ca-125 and Ca-19-9. The sensitivity of Ca-125 is characteristic of the advanced form of the disease, but its specificity is low because it is often elevated in other gynecological diseases [156]. The concentrations of Ca-125 and Ca-19-9 glycoproteins do not give us definitive answers in the differential diagnostic and therapeutic process, but they help in decision-making and direction of management and therefore remain the recommended complementary tool.

Despite the fact that research on biomarkers of endometriosis are still ongoing, there are still no satisfactory results, which makes it impossible to carry out effective laboratory diagnostics used in the diagnosis and monitoring of the treatment of the disease [157].

Some hope for the discovery of the endometriosis marker is carried by studies of microRNAs circulating in the blood [158]. MicroRNAs are small ribonucleic acid molecules about 22 nucleotides long that regulate gene expression by influencing the translation process. MicroRNAs show stability in tissues and can be easily detected in patients’ serum using quantitative methods such as qPCR. Determination of a single microRNA can help distinguish a healthy person from a sick person. However, the greatest prognostic force is the determination of several microRNAs, the expression of which varies in a given disease.

In studies of microRNAs in endometriosis, it has been shown that a certain group of genes is regulated with the participation of short, relatively stable fragments of RNA. MicroRNAs from the let family are among the dominant in endometrial cells.

In the paper of Sahin et al., Let-7b microRNAs have been shown to influence the expression of the ER-α, ER-ß, Cyp19, KRAS 4A, KRAS 4B, and IL-6 genes [146]. Let-7b has a pleiotropic effect in the pathophysiology of endometriosis, affecting the regulation of estrogen levels and the receptor of these growth factors.

Liu et al. suggested that miR449b3p altered expression in endometriosis affects the development of the disease [159].

Altered expression levels of miR-139-5p and miR-375 were observed in the tissues of the ectopic endometrium. These microRNAs may regulate the expression of the transcription factors HOXA9 and HOXA10 and the endothelin 1 gene (EDN1) playing a role in vascular homeostasis, which may be related to the development of endometriosis [160].

Laudański et al. showed that the mammalian target of rapamycin (mTOR) and VEGF pathway genes can be regulated by abnormal miRNA expression in endometriosis [161].

The protein kinase mTOR regulates the process of cell growth, proliferation and movement, as well as translation and transcription processes, while VEGF factor intensifies the process of angiogenesis in endometriosis. A team of Polish researchers conducted miRNA profiling of the eutopic endometrium from women with endometriosis [162]. A sample of 667 human miRNAs were studied in patients with endometriosis compared to the control group. Two of the study miRNAs were elevated, while 13 miRNAs had reduced levels of expression in eutopic endometrium in endometriosis patients compared to control. In addition, hsa-miR-483-5p and miR-629 have been shown to be significantly reduced in expression in endometriosis patients [163].

Gu et al. identified 14 miRNAs that might be involved in the pathogenesis of ovarian endometriosis: hsa-let-7a-5p, hsa-let-7b-5p, hsa-let-7d-5p, hsa-let-7f-5p, hsa-let-7g-5p, has-let-7i-5p, hsa-miR-199a-3p, hsa-miR-320a, hsa-miR-320b, hsa-miR-320c, hsa-miR-320d, hsa-miR-328-3p, hsa-miR-331-3p and hsa-miR-320e.Among them, 10 miRNAs were reported for the first time to be associated with endometriosis [164]. The miRNA hsa-let-7i-5p and hub miRNAs including hsa-let-7a-5p, hsa-let-7b-5p, hsa-miR-320a, and hsa-miR-320d might be potential diagnostic biomarkers [164].

Recent studies showed that the levels of miRNAs 199b-3p, 224-5p, and Let-7d-3p in plasma are potential diagnostic biomarkers for endometriosis patients [165]. In addition, miR-146a, miR-149 and miR-499 may have a role in the pathogenesis of endometriosis [166].

Some miRNAs are associated with genetic, epigenetic and angiogenic factors, hormones, cytokines, chemokines, markers of oxidative stress, mediators of inflammation, hypoxia, angiogenesis, and altered immune systems, contributing to the pathogenesis of endometriosis. Hormonal imbalance occurs by reducing the levels of miRNA-23a and miRNA-23b and increasing miRNA-:135a, 135b, 29c and 194-3p [167]. Angiogenesis by vascular endothelial growth factor is attributed to an increase in miRNA-126, miRNA-210, miRNA-21, miRNA-199a-5p and miRNA 20A. OS upregulates miRNA-302a by increased levels of tumor necrosis factor (TNF)-α, TNF-β and interleukin-1β. Upregulation of miRNA-199a and miRNA-16 promotes inflammation and cell proliferation in endometrial lesions [168]. The gold standard in diagnosing endometriosis is laparoscopy; however, miRNA can be validated as a diagnostic tool for detection, progression and prevention of endometriosis in large, independent cohorts of women, with and without endometriosis.

Despite the identification of several miRNAs, the studies are investigatory in nature. To date, no specific miRNA has been validated for diagnostic purposes (systematic review conducted on PubMed^®^, Latin American and Caribbean Health Sciences Literature (LILACS), MEDLINE^®^ and Web of Science databases using the search terms endometriosis (all fields) AND miRNA (all fields), evaluating all publications up to May 2019) [168].

In conclusion, studies of the diverse microRNA pool in endometriosis have shown that many biological processes are regulated by the above molecules and can have a significant impact on the development of lesions. However, the use of microRNA in the diagnosis of endometriosis is only at the initial stage of research [169,170].

Recently, in the context of endometriosis, long non-coding RNAs (lncRNAs) that cover more than 200 bp and are a subtype of non-coding RNAs (ncRNAs) have become the focus of interest [171].

Unlike a group of short, non-coding RNAs (sncRNAs) such as microRNAs, long non-coding RNAs (lncRNAs) tend to exhibit greater sequence matching and thus specificity of action relative to the target genes. Non-coding RNA molecules participate in the processes of regulation at virtually all stages of the transmission of genetic information: from DNA to protein. Particularly spectacular is the involvement of certain non-coding RNA molecules in the mechanisms leading to the switching on or off of the expression of individual genes.

Zhou et al. found that 388 transcripts of lncRNA tested showed overexpression and 188 showed decreased expression levels in the ectopic endometrium compared to eutopic endometrium [172].

Liu et al. showed that LncRNA H19 may be involved in the pathogenesis of endometriosis especially in the mechanism of recurrence and is a novel potential predictor of the recurrence of endometriosis. In this study LncRNA H19 expression in the ectopic and eutopic endometria of endometriosis patients was significantly higher than that in the normal endometrium [172].

AFAP1-AS1 (AFAP1 Antisense RNA 1) is an RNA Gene, and is affiliated with the lncRNA class. Recent studies have shown that AFAP1-AS1 may be a potential therapeutic target for controlling the progression of endometriosis [161]. AFAP1-AS1 silencing can inhibit cell proliferation and promote apoptosis by regulating the STAT3/TGF-β/Smad signaling pathway by targeting miR-424-5p in endometrial stromal cells [173].

A study by the Cui et al. team explains that LINC01116 promotes the progression of endometriosis through the miR-9-5p/FOXP1 axis. This discovery provides a new therapeutic target for endometriosis patients [174].

HOX antisense intergenic RNA (HOTAIR) is a recently discovered long non-coding RNA (lncRNA) that plays critical role in gene regulation and chromatin dynamics, which appears to be mis-regulated in endometriosis. HOTAIR interacts with key epigenetic regulators such as histone methyltransferase PRC2 and histone demethylase LSD1 and regulates gene silencing.

Research indicates a significant role for HOTAIR in promoting endometriosis [175]. HOTAIR can be used as a potential target for clinical applications. Higher levels of HOTAIR mRNA have been shown in endometrial patients with severe endometriosis.

It is suggested that lncRNAs are closely related to the process of endometriosis. Nevertheless, the molecular mechanisms by which lncRNAs bind to endometriosis need to be explained in more detail. In summary, lncRNAs show potential as biomarkers of endometriosis [176]. However, the clinical significance and biological mechanism of lncRNA in the development of endometriosis remain largely unknown.

## 11. Endometriosis—Genetics

### 11.1. Hereditary Genetic Polymorphism

The concept of the genetic basis for the development of endometriosis derives from many years of observations, in which the family occurrence of this disease was indicated. Since the 1940s, a much higher incidence among women from families in which women suffered from endometriosis has been described, as well as a high incidence among siblings, especially monozygotic twins, where the percentage of co-occurrence exceeds 80% [177]. On the basis of statistical analyses of the family history of endometriosis, it has been proven that a genetic factor is responsible for about 50% of the predisposition to the disease [178]. It is believed that genetic and epigenetic conditions are rather conducive to the development of endometriosis, which is caused by environmental factors, or genetic and epigenetic changes caused by environmental factors are necessary to move from subtle changes in endometriosis to the disease stage [179]. The different phenotypic traits observed among humans are conditioned by millions of polymorphisms scattered throughout the genome, of which single nucleotide polymorphism (SNPs) account for about 90% of the total phenotypic variability. Polymorphic variants sometimes occur in a large percentage of the population and are associated with a variety of phenotypic traits, including susceptibility to various conditions. It has been postulated that certain SNPs systems, perhaps related to jointly inherited haplotypes, may promote the development of endometriosis. In a study on coupling analysis conducted on thousands of families burdened with endometriosis (GWAS–genome-wide association study) from different populations, a number of potential regions associated with the inheritance of this disease were indicated.

The epidemiology of endometriosis can be better understood thanks to the work on the human genome, especially the development of the genome-wide association study (GWAS) technique, i.e., associative studies of the entire genome.

The first GWAS study on endometriosis was published between 2010 and 2011—two papers on the Japanese population and one study of European women [180,181,182].

Studies of the Japanese population showed the relationship of polymorphism rs10965235, in the CDKN2BAS gene at locus 9p21 and rs16826658, in the area of the WNT4 gene at locus 1p36 [183].

Extensive GWAS research was conducted by the International Endo Gene Consortium (IEC) in populations of British and Australian women with endometriosis [165]. On the basis of these studies, loci associated with the development of the disease–7p15.2–was established. This place is among the genes known so far responsible for the development of the uterus and placenta [182].

Of lesser importance in the mentioned populations was the loci of chromosome 1p36 previously announced by Japanese studies [180].

In 2012, an international team of scientists conducted the largest genome-wide association study to date, the first among European women comparing DNA from 5586 women with endometriosis and 9331 people free of the disease [183].

The team identified two regions of the genome associated with an increased risk of endometriosis. The first is located on chromosome 7. This region may be involved in the regulation of nearby genes involved in the development of the uterus and its lining. The second variant is located near the WNT4gene, which is involved in hormone metabolism and the development and functioning of the woman’s genital tract. The important role of the WNT4, CDKN2BAS and FN1 genes was confirmed by the research of the team of Pagliardini et al. [184].

Research indicates a link between the 2p25.1 region, located near the GREB1 gene, and the risk of endometriosis [185].

Literature data on GWAS studies suggest that certain genetic variants, significantly more common in endometriosis, appear to be good functional candidates for the genetic factors responsible for the onset of this disease [185].

GWAS research in endometriosis is still ongoing, providing new results. Literature reports on 998 Belgian patients with endometriosis and 783 controls showed that the polymorphisms rs7521902, rs13394619 and rs6542095 may be associated with this disease [170].

It is worth noting that three genetic variants in the area of GREB1 (close to rs13394619) and CDKN2B-AS1 (close to rs1537377) also showed nominally significant associations with endometriosis [186].

In the paper of Mafra et al., it has been suggested that analysis of genetic variants in the WNT4 gene area (rs3820282, rs16826658) may help identify patients at high risk of developing this disease [187].

Albertsen et al. conducted a GWAS study in the European population of 2019 surgically confirmed cases of endometriosis and 14,471 controls [188].

Three of the single nucleotide polymorphisms (SNPs) associated with the disease have been identified: LINC00339-WNT4 at locus 1p36.12 (rs2235529) and RND3-RBM43 at locus 2q23.3 (rs1519761 and rs6757804). The meta-analysis identified two additional loci that were associated with endometriosis: RNF144B-ID4 at 6p22.3 (rs6907340) and HNRNPA3P1-LOC100130539at 10q11.21 (rs10508881).

In the work of the Polish team Sobalska et al., GWAS analysis found statistically significant links between new SNPs not previously described in the literature and endometriosis [189]. In these studies, a relationship was observed between the rs10129516 polymorphism, located on chromosome 14 in the PARP1P2 and RHOJ intergenic region, and endometriosis. Gen RHOJ encodes one of many small proteins of the Rho family that bind GTP (guanosine-5′-triphosphate), which acts as an energy transporter in the cell. Rho proteins regulate the dynamic assembly of cytoskeleton components in several physiological processes, such as cell proliferation and motility. Rho are also involved in cancer transformation and metastasis. The protein encoded by the RHOJ gene is activated by vascular endothelial growth factor and can regulate angiogenesis and also plays an important role in adipocyte differentiation, endothelial motility, and cytoskeletal formation [190]. Overexpression of the RHOJ gene has been shown in ectopic endometrium [191]. Endometriosis can be the cause of ectopic pregnancy. The risk of such a pregnancy is therefore increased in women with endometriosis. It can therefore be assumed that the RHOJ gene may be involved in the development of endometriosis.

Genetic analyses of the Bylińska et al. confirm the role of polymorphisms of the HLA-G gene and its receptors LILRB1 and LILRB2 for the development of endometriosis [192].

Human leukocyte antigen G (HLA-G) is recognized by KIR2DL4, LILRB1 and LILRB2 receptors on NK cells, antigen-presenting cells, T cells and others. Expression of HLA-G molecules in the ectopic endometrium has been demonstrated. The genes for the receptors KIR2DL4, LILRB1 and LILRB2 are polymorphic, which may affect their activity. The study of Polish scientists analyzed whether polymorphisms of HLA-G, KIR2DL4, LILRB1 and LILRB2 genes may affect susceptibility to endometriosis and disease progression. It was shown that the GG genotype of the rs1632947 polymorphism of the HLA-G gene played a protecting role against disease and its severe stages; similarly, the CT genotype of the polymorphism rs1233334 HLA-G played a protective role against disease progression. The AA genotypes of the rs41308748 polymorphism of the LILRB1 gene and AG rs383369 of the LILRB2 gene predisposed to endometriosis and its progression. No association of polymorphism of the KIR2DL4 gene with endometriosis was observed [193].

The work of another team of Polish researchers showed the relationship of polymorphisms rs12700667 and rs4141819 of the RAF gene with infertility in women with advanced endometriosis [193].

Recent research points to new genes and their polymorphisms related to endometriosis. Christofolini et al. showed a correlation of the polymorphisms rs10928050 of the KAZN gene and the rs2427284 of the LAMA5 gene with endometriosis [194].

Genetic studies provide evidence that changes in DNA increase the likelihood of some women developing endometriosis. Genetic contribution appears to be particularly large in more serious forms of the disease. Many years of GWAS molecular studies have resulted in the selection of several candidate genes as potential markers of endometriosis [195].

The study, which is a systematic review of GWAS studies published by PubMed until December 31, 2019, indicates that variants of the genes WNT4 rs7521902, GREB1 rs13394619, FN1 rs1250248, IL1A rs6542095 and VEZT rs10859871 may affect the development of endometriosis. However, replication and validation of these variants in different populations are essential for a better understanding of endometriosis etiopathogenesis, to optimize diagnosis, and to improve the effectiveness of clinical treatment of the disease [196].

Quite high hopes were associated with the possibility of determining the operation of polymorphisms of genes responsible for the metabolism of hormones and xenobiotics. Most of the variants described in the context of changes in drug metabolism were found to have no effect on the risk of endometriosis. The few polymorphisms associated with this disease include rs12248560 and rs4244285 that alter the expression of CYP2C19, which is involved in the breakdown of estrogens and xenobiotics. Other significant changes concern the deletion variant of the glutathione transferase (GSTM1) gene and the glutathione transferase (GSTP1) variant rs1695, which as phase II detoxification enzymes participate in the final removal of drugs and toxins. The presence of glutathione transferase polymorphisms combined with significant dioxin exposure was associated with the development of endometriosis [197].

A recently published meta-analysis showed that in addition to the above-mentioned variants, gene variants for interferon γ (IFNG CA repeat) and variants of rs16826658 and rs2235529 of the WNT4 gene may be relevant. An unknown trend was also confirmed for the progesterone receptor (PGR) gene variant, the rs1799969 variants of the ICAM1 molecule, rs2292596 of the aromatic hydrocarbon receptor repressor gene (AHRR), the CYP17A1 rs743572 gene and the rs1801282 gene for the peroxisome γ proliferator-activated receptor (PPARγ–peroxisome proliferator-activated receptor γ) [198].

Estrogen receptors, acting as transcription factors, play a significant role in endometrial growth and differentiation, as well as in numerous biological functions in eutopic endometrium and endometriosis. The ERβ receptor encoded by the ESR2 gene is the dominant isoform in those with endometriosis [198].

A different expression of aromatase in endometriosis foci has been demonstrated compared to eutopic endometrium. CYP19A1 is a gene encoding aromatase–an enzyme involved in the biosynthesis of estrogens. The exact basis of the observed changes is not yet known.

Literature data indicate that studies draw attention to the significant role of estrogen receptor genes, especially the ESR2 gene and the CYP19A1 gene for the susceptibility and occurrence of endometriosis [199,200,201]. The study identified statistically significant correlations between the new, previously unseen, two SNPs and endometriosis: rs4986938 and rs928554 [200,202]. In the case of rs4986938, the AA genotype was found to reduce the risk of endometriosis. A similar effect was demonstrated in the presence of the AG genotype of rs928554. The results obtained during the analysis indicate that the polymorphisms rs4986938 and rs928554 of the ESR2 gene are associated with the occurrence of endometriosis. Recent studies have shown that the polymorphisms rs17179740 of the ESR2 gene and rs2899470 of the CYP19A1 gene are associated with the occurrence of endometriosis [202]. In the studied group of women with endometriosis, a significant increase in the expression of the ESR2 gene was found, which may indicate the participation of this factor in the pathogenesis of endometriosis [200]. The results obtained in the work contribute to the broadening of knowledge in the subject of molecular mechanisms conducive to the development of endometriosis. Due to the small amount of research on the analysis of CYP19A1 and ESR2 gene expression and polymorphisms in patients with endometriosis, the research makes a significant contribution to expanding the knowledge about the impact of genetic factors on the development of endometriosis.

Understanding the relationship between gene expression and polymorphism and endometriosis may contribute to the development of new therapeutic strategies in the context of this disease. Molecular studies may be a target for personalized therapy in the future.

### 11.2. Somatic Mutations

The association of endometriosis with cancer, which appears as a result of genetic mutations, contributed to the search for pathogenetic mutations occurring in cells during the development of the disease. The concept of this research is based on the hypothesis that at least some of the changes arise as a result of somatic mutations absent in the germline and occur during the development of an individual organism. The occurrence of de novo mutations in both the endometrial cyst epithelial and peritoneal foci as well as in DIE foci is described. The most frequently noted genes are: ARID1A, KRAS, PIK3CA and PPP2R1A [203,204].

Analyzing samples taken from DIE patients with the help of next generation sequencing (NGS), it was possible to describe 51 new mutations involving a large group of proto-oncogenes. Some of these mutations have previously been described as mutations that have an initiating effect on the formation of tumors. The new mutations can affect both the eutopic endometrium and endometriosis foci, and do not have to occur in the germline cell line. They can also selectively appear only in the foci of endometriosis themselves. The location of the mutation in the genome appears to be specific to the patient and is not repeated in others. It is emphasized that the impact of these mutations on the development of the disease is complex and requires further evaluation. However, it can be assumed that these patients will be burdened with a higher risk of cancer in the future [203].

## 12. Summary

Analyzing the research of recent years on endometriosis, it can be seen that the current therapeutic options available are not optimistic. Unfortunately, the treatment of this disease is still ineffective. However, the progress of knowledge, especially at the genetic level, which has taken place in recent years, allows the separation of certain molecular shields for new therapeutic methods. Much hope is associated with new directions of research, such as the use of miRNAs or lncRNAs, which regulate key cellular pathways in the development of the disease, as molecular markers in endometriosis. However, these studies must involve large groups of patients with their full clinical description in order to be able to draw conclusions about the use of these particles as markers of endometriosis. There are reports of the development of the possibility of using ncRNA as diagnostic tools [205]. It remains to be seen whether genetic research will allow for the establishment of new therapeutic regimens and significantly contribute to improving the treatment results of women suffering from endometriosis.

## Figures and Tables

**Table 1:** Theories of the formation of endometriosis—a historical outline.

Name of the Investigator	Year	Theory
Recklinghausen	1885	from Wolff wires
Cullen	1896	from Müller cables
Iwanhofen	1898	metaplasia theory—the assumption of the theory is the existence of cells capable of differentiating in the endometrium, and being precursors of the epithelium of the pelvic peritoneum
Meyer	1903	metaplasia theory—the assumption of the theory is the existence of cells capable of differentiating in the endometrium, and being precursors of the mesodermal epithelium of the ovary
Pick	1905	metaplasia theory; regarding the sexual epithelium of the ovary
Sampson	1927	“retrograde menstruation”
Halban	1924	endometrial elements can enter the peritoneal cavity by the blood or lymphatic route
Navrital i Kramer	1936	vascular spread
Javert	1949	a combination of implantation theory, transport by the blood and lymphatic routes, as well as the theory of direct penetration of the endometrium through the uterine muscle
Mc Weigh	1955	from the cells of the radial wreath of the egg
Weed i wsp	1980	failure of the immune system derived from the Müller ducts
Malick	1982	congenital or acquired weakened fibrinolytic activity of the peritoneum

**Table 2:** Cytokines associated with the development of endometriosis.

Immune Factor	Role in Endometriosis
TNF-α	Increasing vascular permeability and transformation of inflammatory factors in the peritoneal cavity, which exacerbate peritonitis [66].
NF-κB	Controlling gene expression associated with immune response, cellular proliferation, and cytokine production [67].
MCP-1	Stimulation of monocytes to migrate from peripheral blood to the peritoneal cavity to turn into macrophages, leading to local inflammation [68].
IL-1β	Induction of VEGF and COX-2 expression leading to the progression of endometriosis [69].
IL-6	In the tide to impair the function of NK cells (natural *killer)* by regulating the protein expression of tyrosine phosphatase (SHP-2) in endometriosis [70].
IL-10	In a mouse model with induced endometriosis, inhibition of IL-10 activity was found to be helpful in reducing lesions [71].
IL-15	In normal endometrial cells, ovarian steroid hormones control the production of IL-15. Endometriotic cells in patients with endometriosis show higher concentrations of this cytokine in endometrial patients [72].
IL-16	Higher concentrations of IL-16 in women with endometriosis are associated with the development of the disease by stimulating the secretion of IL-6, TNF-α and IL-1β. IL-16 polymorphisms are associated with women’s susceptibility to the development of endometriosis and its severity [73].
IL-17A	In endometriosis, IL-17A is expressed in endometrial lesions, and therefore the inflammatory environment of the peritoneal cavity of patients with endometriosis may be associated with the production of IL-17A [74].
IL-18	IL-18 regulates the production of TNF-α and IL-8, acts as a potent angiogenic factor, and also regulates the intercellular expression of adhesion molecule 1 through NFκB and may increase MMP production. IL-18 is a major regulator of the immune response process in a wide range of cells that decreases in both eutopic and ectopic endometrium in endometriosis [75].
IL-27 IL-33	IL-10 + Th17 stimulate the proliferation and implantation of ectopic lesions and accelerate the progression of endometriosis, making IL-27 a key regulator in endometriotic lesions [76]. Member of the IL-1 family. IL-33 induces the synthesis of Th2-type cytokines through its orphan receptor ST2. Increased IL-33 expression has been correlated with fibrotic disorders such as skin scleroderma, liver and lung fibrosis, making IL-33 a key profibrotic mediator [77].
IL-37	Increased levels of IL-37 expression in eutopic and ectopic endometrium in women with stage III-IV ovarian endometriosis may be involved in inflammatory processes leading to endometriosis [78].

**Table 3:** Types of divisions of endometriosis by location.

According to the Classical Classification of Martius	According to Kistner’s Classification
- endometriosis genitalis interna (adenomyosis) is present in the uterus or the Fallopian tube - endometriosis genitalis externa (in the remaining parts of the reproductive organ) - endometriosis extragenitalis (endometriotic lesions are present outside the reproductive organs)	1. Overlapping peritoneal endometriosis Ovaries serous membrane of the uterus uterine ligaments Fallopian tubes large intestine, thin intestine, appendix 2. Retroperitoneal endometriosis inguinal regio neck, vagina, vulva, perineum drainage pathways pleural and lungs skin, skeletal muscles, limbs

**Table 4:** Groups of medicines used in the treatment of endometriosis.

	Types of Drugs
1.	non-steroidal anti-inflammatory drugs
2.	hormonal drugs: progestogens hormonal contraceptives, danazol, analogues and gonadoliberin (GnRH)–gonadoliberin agonists and antagonists
3.	selective progesterone receptor modulators
4.	aromatase inhibitors

## Data Availability

Not applicable.
